# Applying Advanced Practice Nurses in Rural Japan: A Mixed-Methods Perspective

**DOI:** 10.7759/cureus.57015

**Published:** 2024-03-27

**Authors:** Ryuichi Ohta, Satoko Maejima, Chiaki Sano

**Affiliations:** 1 Community Care, Unnan City Hospital, Unnan, JPN; 2 Nursing, Unnan City Hospital, Unnan, JPN; 3 Community Medicine Management, Shimane University Faculty of Medicine, Izumo, JPN

**Keywords:** healthcare disparities, patient care management, electronic health records, curriculum development, advanced practice nursing, rural health services

## Abstract

Introduction

Rural hospitals, particularly those in geographically isolated regions like Shimane Prefecture, Japan, face significant healthcare delivery challenges. These include limited resources, an aging population, and a scarcity of healthcare professionals. Advanced practice nurses (APNs) have emerged as pivotal in addressing these gaps, offering specialized patient assessment, diagnosis, and management skills. This study aimed to evaluate the demand for APNs in rural community hospitals, focusing on the specific educational needs and clinical competencies required to improve healthcare outcomes in these settings.

Method

Employing a mixed-methods approach, this research combined qualitative insights from stakeholder interviews with quantitative data analysis of electronic health records (EHRs) at Unnan City Hospital. This sequential exploratory design aimed to capture comprehensive educational needs and outcomes, integrating the depth of qualitative data with the breadth of quantitative evidence to tailor a curriculum for APNs in rural healthcare contexts.

Results

The study revealed a critical demand for APNs skilled in managing common medical issues in rural settings, such as infections, circulatory failures, and respiratory problems. Stakeholder interviews highlighted the necessity for a curriculum that enhances clinical competencies and emphasizes soft skills like communication and leadership. An analysis of EHRs identified 21 specific diseases across six categories, underlining the importance of targeted education on these prevalent conditions.

Conclusion

The findings underscore the urgent need for specialized education programs for APNs in rural hospitals to address these communities' unique healthcare challenges. Developing a curriculum that focuses on clinical and soft skills essential for rural healthcare delivery can significantly enhance the quality of care. This study advocates for implementing such tailored educational programs to empower APNs, thereby contributing to healthcare equity and improving patient outcomes in rural settings.

## Introduction

Rural hospitals face significant challenges, including limited healthcare resources and aging populations with complex needs [1]. These issues are particularly acute in areas like Shimane Prefecture, Japan, with hospitals such as Unnan City Hospital serving geographically isolated communities [2,3]. The scarcity of healthcare professionals and resources necessitates innovative care strategies, underscoring the critical role of advanced practice nurses (APNs). These nurses, with specialized skills in assessment, diagnosis, and disease management, are pivotal in improving healthcare outcomes in rural settings [4,5].

APNs are vital in bridging healthcare delivery gaps caused by physician shortages and limited resources [6]. Their expertise enhances patient care and supports community health through education on disease management and prevention [7]. However, their impact is contingent upon receiving education, preparing them for rural healthcare's unique challenges. Traditional nursing programs often fall short in this regard, highlighting the need for specialized curriculum development tailored to rural healthcare's intricacies [8,9].

This study aims to evaluate the demand for APNs in rural community hospitals, targeting the specific needs of rural healthcare. By integrating evidence-based practices with practical skills, the curriculum can be developed to equip nurses with the competencies to effectively manage the health needs of rural populations, focusing on aging demographics and common diseases based on this research [10]. The significance of clarification of the demand for APNs in rural community hospitals extends beyond immediate healthcare improvements in rural hospitals. It aims to enhance the capabilities of APNs, contributing to healthcare equity and accessibility.

## Materials and methods

Methodology

The study adopted a mixed-methods approach to comprehensively address the research aim of evaluating the demand for APNs in rural community hospitals. This methodology was chosen for its ability to provide a holistic understanding of educational needs and outcomes by integrating both the depth of qualitative insights and the breadth of quantitative data [11]. The mixed-method approach facilitates the exploration of complex phenomena such as educational interventions, examining how such interventions are implemented and received in real-world settings and how they can be optimized to improve healthcare outcomes in rural areas.

Study design

The research was structured around a sequential exploratory design, starting with qualitative methods to gather in-depth insights into the specific needs, preferences, and experiences of APNs and other stakeholders. This qualitative phase informed the demand for APNs among stakeholders in a rural hospital. Following the qualitative phase, a quantitative approach was used to evaluate the concrete contents for APNs working in rural hospitals. By combining qualitative and quantitative methods, the study aimed to ensure that the working contents were evidence-based and tailored to the unique context of rural healthcare settings.

Setting and participants

Unnan City Hospital in Shimane Prefecture, Japan, was selected as the setting for this study due to its representative nature of rural healthcare challenges, including an aging population and limited access to specialized healthcare services. The hospital is critical to the local healthcare system, making it an ideal site for implementing and evaluating an APN education curriculum [12-14]. Participants were recruited from the hospital staff, including the hospital dean, the nursing director, APNs, and other healthcare professionals interacting with APNs. These stakeholders were chosen for their diverse perspectives and experiences, which are crucial for understanding the multifaceted nature of APNs in rural settings [14]. Their involvement ensured that a comprehensive understanding of nursing practice's clinical, educational, and administrative aspects informed the curriculum development [15]. In total, 16 participants agreed to participate in semi-semi-structured interviews.

Data collection methods

Data collection was conducted in two phases, aligning with the mixed-method design. In the qualitative phase, semi-structured interviews were conducted with selected participants. These interviews explored participants' views on the current state of APN education, perceived gaps in knowledge and skills, and suggestions for curriculum content and delivery. A purposive sampling strategy was employed to ensure a wide range of perspectives. Simultaneously, researchers gathered quantitative data on the demand for APNs regarding specific medical conditions. Electronic health records (EHRs) were analyzed to identify common conditions treated at the hospital and the specific tasks performed by APNs, providing a data-driven foundation for curriculum development.

Data analysis

Qualitative data from interviews were transcribed verbatim and subjected to thematic analysis [16]. This process involved coding the data to identify recurring themes and patterns related to APN education needs, preferences for learning formats, and perceived barriers to effective learning. The analysis was iterative, with codes and themes refined through discussion among the research team to ensure reliability and validity.

The analysis of EHR data involved extracting information on the frequency and types of conditions treated by APNs. This information was used to identify critical areas of clinical practice that APNs should address. Statistical analysis of EHR data helped to prioritize these areas based on their relevance to patient care in the rural setting of Unnan City Hospital.

Ethical considerations

The Unnan City Hospital Clinical Ethics Committee approved the study protocol (no. 20180013).

## Results

The mixed-methods approach employed in this study yielded comprehensive insights into the demands of APNs in rural hospitals. Through interviews and analysis of EHRs, we identified vital stakeholder perspectives and assessed rural hospitals' needs for APNs in rural settings.

Stakeholder insights

Interviews with hospital stakeholders, including APNs, the nursing director, and the hospital dean, revealed several key themes regarding the role of APNs and their educational needs. In total, 16 stakeholders participated in this phase. Through the thematic analysis of the semi-structured interviews for stakeholders, three themes developed: rural hospital needs for advanced care nurses, demand for a comprehensive skillset, and detecting common medical issues for education (Table 1).

**Table 1:** Themes and concrete excerpts APN: advanced practice nurse

Theme	Concrete excerpt
Rural hospital’s needs for advanced practice nurses	“The present rural medical system lacks physicians. They need a longer duration of education. APNs should be educated to satisfy present medical needs in rural hospitals” (Participant B). “Mainly common symptoms should be approached by APNs supporting busy physicians. The common symptoms should be listed for the education of APNs” (Participant A).
Demand for comprehensive skillset	“APNs should communicate with physicians and nurses to avoid conflicts in patient care. They should recognize the present culture of other medical professionals and collaborate with them, respecting their culture” (Participant D). “APNs should take some leadership in care management regarding APN care. In the cases in which they are involved, their effective leadership can improve patient care quality by collaborating with other medical professionals” (Participant F).
Detecting common medical issues for education	“The education for APNs should be focused on specific and common medical issues” (Participant F). “Common medical issues should be based on local medical problems in the community hospital and rural healthcare needs” (Participant I).

Rural Hospital Needs for Advanced Care Nurses

Stakeholders unanimously recognized the critical role of APNs in filling healthcare delivery gaps, particularly in areas affected by physician shortages and limited resources. One of the participants stated, “The present rural medical system lacks physicians. They need a longer duration of education. APNs should be educated to satisfy present medical needs in rural hospitals” (Participant B). Another participant stated, “Mainly common symptoms should be approached by APNs supporting busy physicians. The common symptoms should be listed for the education of APNs” (Participant A). As supporters of physicians, APNs were demanded and needed to be educated based on the needs of rural hospitals, such as approaches to common symptoms.

Demand for Comprehensive Skillset

Stakeholders emphasized the need for APNs to possess comprehensive skills in patient assessment, diagnosis, and management of common diseases, alongside soft skills such as communication and leadership. One of the participants stated, “APNs should communicate with physicians and nurses to avoid conflicts in patient care. They should recognize the present culture of other medical professionals and collaborate with them, respecting their culture” (Participant D). Another participant stated, “APNs should take some leadership in care management regarding APN care. In the cases in which they are involved, their effective leadership can improve patient care quality by collaborating with other medical professionals” (Participant F). APNs in rural hospitals were demanded for leadership and collaboration skills, as well as respect for the cultures of other medical professionals.

Detecting Common Medical Issues for Education

Educational needs identified through interviews included a strong emphasis on practical, hands-on training in clinical settings and theoretical knowledge that underpins clinical decision-making. One of the participants stated, “The education for APNs should be focused on specific and common medical issues” (Participant F). Another participant stated, “Common medical issues should be based on local medical problems in the community hospital and rural healthcare needs” (Participant I). Stakeholders highlighted the importance of a flexible and adaptable curriculum to meet the changing needs of rural healthcare settings and promote lifelong learning and professional development.

The analysis of the electrical record

We used the data about the diseases that physicians treated in a year from the electronic database of our hospital according to ICD 10 (International Classification of Diseases 10). We calculated the numbers of each condition and ordered the diseases based on the number from top to bottom, and the diseases ranked first to 20 were used. From this list, we deleted the ones related to chronic diseases (Table 2). 

**Table 2:** The result of the analysis of the common medical issues in the community hospital

ICD10	Diagnosis	Cases	Category	Total case	Percentage
J159	Bacterial pneumonia	23	Approach to infection	122	26.2
J189	Pneumonia	11			
J690	Aspiration pneumonia	19			
A419	Septic shock	35			
N390	Urinary tract infection	11			
N12	Pyeronephritis	8			
N10	Acute pyeronephritis	15			
E86	Dehydration	61	Diagnosis of circulatory failure	88	18.9
I500	Congestive heart failure	15			
R579	Acute circulatory failure	12			
K590	Constipation	79	Approach to constipation	79	17.0
F059	Delirium	11	Diagnosis of unconsciousness	55	11.8
R402	Unconsicousness	44			
J969	Respiratory failure	9	Diagnosis of respiratory failure	54	11.6
J960	Acute respiratory failure	13			
R090	Hypoxemia	32			
G470	Insomnia	27	Miscellaneous	67	14.4
R33	Uninary retension	12			
A09	Acute gastroenteritis	11			
L309	Exema	9			
E876	Hypokalemia	8			

Table 1 provides a detailed breakdown of common medical issues in a community hospital based on a year-long analysis of electronic health records categorized according to the ICD-10 classification. It highlights six major categories of conditions: approach to infection, diagnosis of circulatory failure, approach to constipation, diagnosis of unconsciousness, diagnosis of respiratory failure, and miscellaneous, with 21 specific diseases identified. The table showcases the frequency of each condition, ranging from bacterial pneumonia to dehydration and acute respiratory failure to insomnia, reflecting the diversity of acute health issues managed in the hospital setting. Notably, conditions like bacterial pneumonia and dehydration appear as prevalent issues, with 122 cases in the infection category and 88 in circulatory failure, indicating significant areas for healthcare focus. This concise organization of data not only reveals the primary health challenges faced but also aids in prioritizing training and resources for APNs to improve patient care outcomes effectively.

## Discussion

The demand for APNs in rural community hospitals has been increasingly recognized as critical to addressing these settings' unique healthcare delivery challenges. Our study, conducted at Unnan City Hospital in Shimane Prefecture, Japan, underscores the pivotal role of APNs in bridging the gap caused by physician shortages and limited healthcare resources. The comprehensive analysis of EHRs and stakeholder interviews revealed significant insights into the educational needs and clinical competencies required for APNs in rural hospitals.

The analysis of EHR data highlighted a spectrum of common medical issues, predominantly infections, circulatory failures, respiratory problems, and other acute conditions, which are prevalent in the rural setting of the study. This finding aligns with existing literature emphasizing the burden of acute conditions in rural areas, where limited access to specialized care and delayed presentations are standard [17,18]. The significant presence of conditions like bacterial pneumonia, dehydration, and acute respiratory failure in our study highlights the crucial roles APNs can play in improving patient outcomes, which can be achieved through early detection, management, and timely referral to specialized care for these conditions [19,20].

Stakeholder interviews further illuminated the expectations and perceived gaps in the current training of APNs. A recurrent theme was the need for a curriculum that equips nurses with advanced clinical skills and emphasizes soft skills such as communication, leadership, and the ability to work collaboratively within multidisciplinary teams [21]. This is particularly relevant in rural healthcare, where APNs often serve as patients' primary point of contact and play a central role in coordinating care [22]. The emphasis on soft skills underscores the complexity of rural healthcare delivery, where APNs can navigate diverse patient needs, scarce resources, and cultural sensitivities.

Our findings suggest a significant gap between the current educational offerings for APNs and the specific needs of rural healthcare settings. While most training programs provide a solid foundation in clinical skills, more focus needs to be placed on rural healthcare's unique challenges, such as managing a wide range of conditions with limited resources and the importance of building strong community relationships [23,24]. This gap highlights the need for evidence-based curriculum development that is contextually adapted to the rural healthcare landscape.

The call for a specialized curriculum for APNs in rural settings is familiar; however, our study provides concrete data to support this demand and outlines the specific areas of focus that such a curriculum should address [25]. By integrating the practical skills required to manage common medical issues identified in our study with an emphasis on soft skills, the proposed curriculum aims to prepare APNs to effectively meet the unique demands of rural healthcare delivery [26].

This study's primary limitation is its focus on a single rural hospital in Unnan City, which may limit the generalizability of the findings to other rural healthcare settings with different demographics, resources, and healthcare challenges. Additionally, the reliance on self-reported data through questionnaires and interviews may introduce response bias, affecting the accuracy of the needs assessment and stakeholder insights. Future research should consider a broader range of rural hospitals and incorporate objective measures of educational outcomes to validate the curriculum's effectiveness across diverse rural healthcare environments.

## Conclusions

The demand for APNs in rural community hospitals is clear and pressing. The development and implementation of a specialized curriculum based on the findings of this study represent a crucial step toward addressing the healthcare disparities rural communities face. Educational institutions, healthcare providers, and policymakers need to collaborate to support the roll-out of this curriculum, ensuring that APNs are well-prepared to meet the unique healthcare needs of rural populations, ultimately contributing to improving healthcare equity and accessibility.
